# Effect of ionic radii on the Curie temperature in Ba_1-x-y_Sr_x_Ca_y_TiO_3_ compounds

**DOI:** 10.1038/srep28055

**Published:** 2016-06-21

**Authors:** A. Berenov, F. Le Goupil, N. Alford

**Affiliations:** 1 Department of Materials, Imperial College London, London, SW7 2AZ, UK

## Abstract

A series of Ba_1-x-y_Sr_x_Ca_y_TiO_3_ compounds were prepared with varying average ionic radii and cation disorder on A-site. All samples showed typical ferroelectric behavior. A simple empirical equation correlated Curie temperature, *T*_*C*_, with the values of ionic radii of A-site cations. This correlation was related to the distortion of TiO_6_ octahedra observed during neutron diffraction studies. The equation was used for the selection of compounds with predetermined values of *T*_*C*_. The effects of A-site ionic radii on the temperatures of phase transitions in Ba_1-x-y_Sr_x_Ca_y_TiO_3_ were discussed.

## Introduction

Perovskites with the general formula: Ba_1-x-y_Sr_x_Ca_y_TiO_3_ (BSCT) show enhancement of several properties (e.g dielectric, piezoelectric^1^, electrocaloric^2^ response) in the vicinity of Curie temperature, *T*_*C*_, where a cubic to tetragonal phase transition occurs and could be strong contenders for Pb-free ferroelectric materials^3^. As a result, the ability to optimize chemical composition of BSCT compounds in order to shift *T*_*C*_ to the desired operating temperature range is important from a practical point of view. Several decades of research have established some of the key parameters that influence the *T*_*C*_ in perovskites: chemical composition of A- and B-sites in ABO_3_ perovskites^4^, A to B nonstoichiometry ratio^5^, lattice parameters^6^, tolerance factor^7^, average mass of A-site ions^8^, ^18^O/^16^O isotope ratio^9^, grain size^10^, annealing temperature^11^, hydrostatic pressure^12^, strain in thin film^13^. Nevertheless there is still a lack of simple guidelines for the selection of the materials compositions with predetermined value of *T*_*C*_.

The average ionic radius, <*r*_*A*–*site*_>, of A-site ions has a strong effect on the *T*_*C*_ especially in Ba_1-x_Sr_x_TiO_3_ compounds where *T*_*C*_ decreases linearly with Sr doping. This decrease is usually explained by the substitution of Ba ions by smaller Sr ions resulting in the observed linear decrease of the *T*_*C*_ with lattice parameter in Ba_1-x_Sr_x_TiO_3_ (*a* or 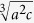) for cubic or tetragonal phases, respectively)^6^. Similarly, the decrease *T*_*C*_ with applied hydrostatic pressure^12^ was explained by the pressure induced decrease of the unit cell volume^14^.

Another parameter affecting *T*_*C*_ is the A-site cation disorder which can be quantified by variance, σ^2^, as follows^15^


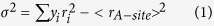


where *r*_*i*_ and *y*_*i*_–ionic radii and occupancy of A-site of element i, respectively. The linear increase of the *T*_*C*_ with σ^2^ in BSCT has been observed when the average ionic radius was kept constant^16^. In other perovskite or perovskite related structures, the increase of σ^2^ was shown to decrease the metal-insulator transition temperature in manganites^15^ and decrease critical current in YBCO-type superconductors^17^. In this work we evaluated the effects of average ionic radius (ionic radii in 12 fold coordination were used^18^) and ionic radii variance of A-site ions in the perovskite lattice on the phase transitions in BSCT compounds.

A series of compounds (see Table 1) were prepared by a conventional solid-state reaction synthesis with starting chemicals BaCO_3_ (Alfa Aesar, 99.95%), CaCO_3_ (Alfa Aesar, 99.95%), SrCO_3_ (Alfa Aesar, 99.99%) and TiO_2_ (PI-KEM Ltd., 99.9%) calcined at 1300 °C for 10 hrs. Three series of compounds were prepared: series A where <*r*_*A*–*site*_> was fixed at 1.551 Å and σ^2^ was varied from 0.0066 to 0.0125 Å^2^, series B where both <*r*_*A-site*_> and σ^2^ were varied, series C where both <*r*_*A-site*_> and σ^2^ were varied in order to maintain constant *T*_*C*_ as described below. Chemical composition was confirmed by ICP-OES analysis (DV 200 OES, Perkin Elmer). XRD (X’PERT MPD, PANalytical) showed that all specimens were single phase tetragonal perovskites (4 mm s.g.) at room temperature except for Ba_0.78_Ca_0.22_TiO_3_ where a weak peak belonging to CaTiO_3_ was observed (see Supplementary Fig. S1). This composition is close to the temperature dependent Ca solubility limit of 16% at 1300 °C^19^, 25% at 1430 °C^20^ and 1549 °C^21^. A good agreement between the values of the lattice parameters of Ba_1-x_Sr_x_TiO_3_ with those reported in the literature^22^ was observed. For the series A the increase of variance resulted in a slight decrease of the a lattice parameter and increase of the c lattice parameter whereas the c/a ratio showed an increase. Dense pellets (>92% dense) were prepared by sintering at 1450 °C. The average grain size was 30–65 μm as observed by SEM (JSM 6400, JEOL) and shown in Supplementary Fig. S2. DSC measurements (DSC200 F3, Netzch) during 5 K/min heating showed well defined peaks at *T*_*R-O*_, *T*_*O-T*_ and *T*_*C*_ which correspond to consecutive phase transitions during heating from rhombohedral (3 m) to orthorhombic (mm2) to tetragonal (4 mm) and, finally, to cubic (m3 m) structures, respectively (see Supplementary Fig. S3 and Supplementary Table S4).

**Figure 1:**
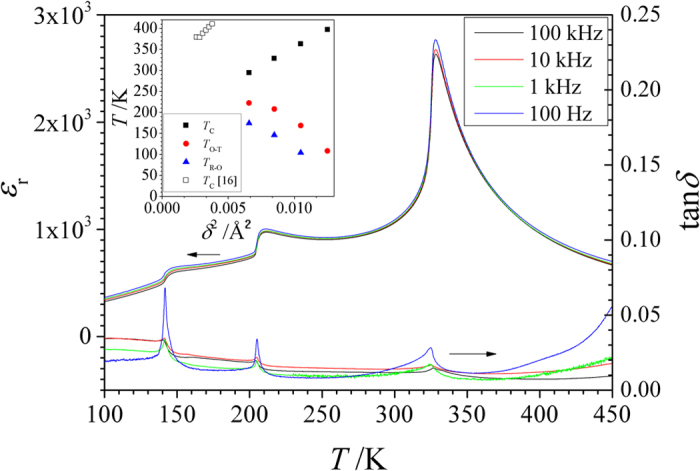
Temperature dependence of permittivity and loss tangent of Ba_0.69_Sr_0.24_Ca_0.07_TiO_3_ at 0.1, 1, 10 and 100 kHz. Measurements were performed during 1 K min^−1^ heating. Inset shows temperatures of phase transitions in BSCT compounds (series A) as a function of *σ*^2^.

The dielectric properties were evaluated by HP 4263B LCR with 0.3 V/mm ac signal at 0.1–100 kHz. The thermal hysteresis observed between heating and cooling runs was less than 3 K. Dielectric permittivity, *ε*_*r*_, peaked at *T*_*C*_ and showed humps at *T*_*R-O*_ and *T*_*O-T*_ (Fig. 1). No frequency dependence of *ε*_*r*_ was observed. The tanδ showed clear peaks positioned slightly below (by less than 10 K) the temperatures of the corresponding phase transitions. The estimated error in the determination of temperatures of phase transitions is 1.5 K and a good agreement was observed between the values of *T*_*C*,_ *T*_*R-O*_ and *T*_*O-T*_ determined from DSC and LCR measurements. Both *T*_*R-O*_ and *T*_*O-T*_ decreased with Ca doping and no *T*_*R-O*_ and *T*_*O-T*_ were observed in the samples with more than 20 and 15% of Ca doping, respectively, in agreement with the literature^23^,^24^. *T*_*C*_ increases linearly with the tetragonal distortions in the lattice (expressed as c/a ratio) regardless of the chemical composition of samples (Supplementary Fig. S5). The ε_r_ data located 10–20 K above *T*_*C*_ were fitted to the Curie-Weiss law (Table 2). The value of *T*_*0*_ determined from the fitting, was smaller than *T*_*C*_ suggesting a first order phase transition in all studied samples.

It has been shown that the increase of cation disorder results in the formation of relaxor type behavior^25^. As a result a modified Curie-Weiss law^26^ was used to fit the data


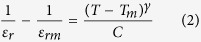


where ε_rm_ is the permittivity at *T*_*C*_, γ is exponent which is expected to be 1 for classical ferroelectrics and 2 for relaxors. Relatively low values of γ measured in this work (1.03–1.27), the lack of frequency dependence of ε_r_ and the closeness of the peaks on ε_r_ and δ temperature curves, suggested that all compounds investigated showed typical ferroelectric behavior. The linear increase of the *T*_*C*_ with the σ^2^ was observed (inset Fig. 1). The observed 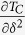 slope was slightly larger (17252 K Å^−1^) than previously reported (14500 K Å^−1^) for BSCT compounds with <*r*_*A*−*site*_>  = 1.594 Å^16^. *T*_*T-O*_ and *T*_*R-O*_ showed a monotonic decrease with σ^2^.

**Figure 2:**
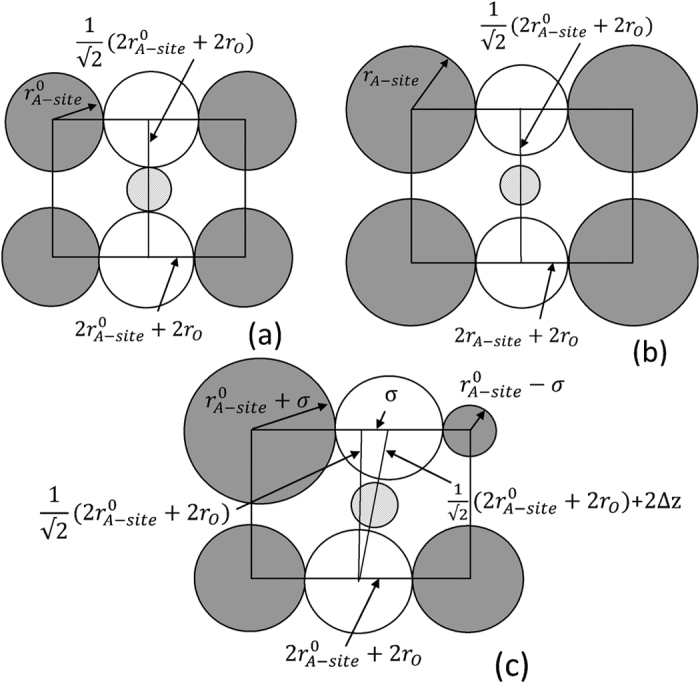
Schematic representation of (110) planes in the ideal cubic perovskite with 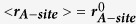 and σ^2^ = 0 (**a**), in the cubic perovskite with 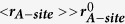 and σ^2^ = 0 (**b**) and in the cubic perovskite with 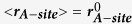 and σ^2^ > 0 (**c**). Grey, white, and patterned circles represent A-site ions, O and Ti ions, respectively.

Recently a combined effect of the average ionic radii and cation variance on the phase transition temperature in perovskite (alkaline earth doped rare earth manganites) and perovskite related (cuprate superconductors) compounds was proposed^27^. For example, a linear decrease of the metal-insulator transition temperature with the increase of the 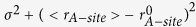 function was observed in a large number of manganites^28^. 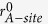 is the ionic radius of the “ideal” non-distorted cubic perovskite which can be calculated from geometrical considerations as 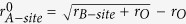 where *r*_*B*−*site*_ and *r*_*O*_ are ionic radii of B and O ions in ABO_3_ perovskite, respectively. An empirical hard sphere ionic model has been proposed^28^ where the increase in σ^2^ and 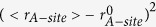 determined BO_6_ octahedra tilting in perovskites with the tolerance factor, *t*, less than 1 and introduced strain like energy term affecting the temperature of phase transition. Similar hard sphere ionic model was applied to the perovskites studied in this work with the *t* > 1. In this case the lattice distortions caused by ion size mismatch relieved not by BO_6_ octahedra tilting but by shifting of oxygen ions leading to the distortion of BO_6_ octahedra^29^. In the model the ferroelectricity was assumed to be caused by the shift of Ti ions in the direction of equatorial oxygen in TiO_6_ octahedra however the results of local-structure refinements in Sr doped BaTiO_3_ suggested 4 site distribution of Ti ions in tetragonal and 8 site distribution in cubic phases with the site splitting of ≈0.2 Å^29^,^30^. In the ideal non-distorted cubic perovskite (*t* = 1) Ti and O ions were closely packed and no shift of Ti ions from the centrosymmetric positions were possible (Fig. 2(a)). An increase of <*r*_*A*–*site*_> by the introduction of larger A-site ions (σ^2^ = 0 Fig. 2(b)) or an increase of σ^2^ by the introduction of cation disorder (<*r*_*A*–*site*_>  = 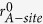 Fig. 2(c)) was likely to enlarge TiO_6_ octahedra and allowed Ti ions to shift from the centrosymemtric position at T < *T*_C_ by the distance *d*. From the geometrical considerations:


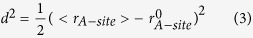


and (assuming 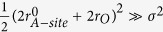)


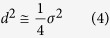


for the former (Fig. 2(b)) and later (Fig. 2(c)) cases, respectively. It has been shown both empirically^31^ and theoretically^32^,^33^ that *T*_*C*_ depends on the atomic displacement of B-site cations, *d*, as *T*_*C*_ ~ *d*^2^.

As a result an increase of the *T*_*C*_ with both 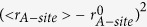 and *σ*^2^ was expected. However a poor correlation of values of *T*_*C*_ with 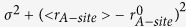 function was observed for the samples studied in this work (Supplementary Fig. S6) presumably due to the assumptions used in the suggested simple model (spherical non polarizing ions, cubic perovskite structure in the ferroelectric phase, neglect of off centered Ca ions, etc.) and a modified empirical function 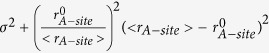 was suggested.

**Figure 3:**
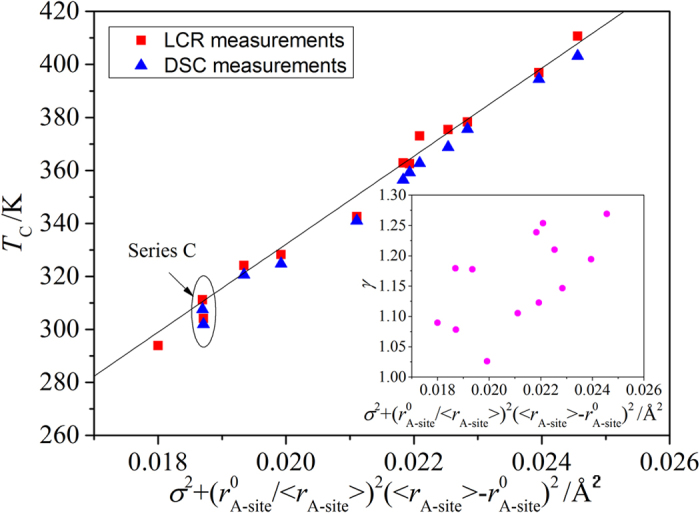
T_C_ as a function of 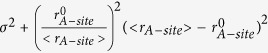. Solid line is a fit to equation 3. Inset shows γ as function of 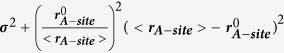.

The *T*_*C*_ of all studied samples showed a linear correlation with the modified function regardless of the cation composition on A-site (Fig. 3) as follows:


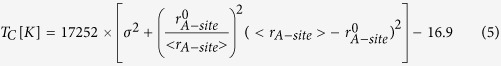


Although the proposed simple model appeared to qualitatively explain the empirical relationship of *σ*^2^ and <*r*_*A*−*site*_> with *T*_C_, a detailed model is required to relate ionic radii of individual ions with the following factors influencing ferroelectricity in titanate perovskites: magnitude of Ti off centering in TiO_6_ octahedra, the direction of the Ti off centering and contributions from A-site ions displacements. Recent studies of local structure provided vital information on the influence of Ca and Sr substitution in BaTiO_3_ on the above mentioned parameters^29^,^30^,^34^. The magnitude of Ti off centering was found to decrease monotonically with Sr doping in BaTiO_3_ as TiO_6_ octahedra became more regular and reduced in volume^29^,^30^,^35^. This effect is usually associated with the observed decrease of *T*_C_ upon Sr doping. The effect of Ca doping on TiO_6_ octahedra remains controversial. It was shown that Ca doping decreased the average volume of TiO_6_ octahedra whereas the degree of distortions in TiO_6_ network increased^35^. The volume of some TiO_6_ octahedra was found to be close to the one of BaTiO_3_ even when 30% Ca was introduced on A-site. On the other hand the increase of the volume of TiO_6_ octahedra with the increasing number of neighboring Ca ions and concurrent increase of the Ti displacement was reported^30^. The direction of Ti ions displacement was found to be aligned at ≈33° with respect to the c axis in BaTiO_3_^29^. Sr doping increased this angle of alignment to 39° in Ba_0.8_Sr_0.2_TiO_3_ and 54° (Ti displacement along (111) direction) in Ba_0.5_Sr_0.5_TiO_3_. As a result the c-axis component of polarization diminished lowering the value of *T*_C_. Ca doping appeared to induce an opposite effect as the direction of Ti ions displacement was closely aligned with c axis in Ba_0.7_Ca_0.3_TiO_3_^30^. No Sr off-centering was observed in Ba_1-x_Sr_x_TiO_3_ materials resulting in an isotropic relaxation of oxygen ions around the A-site thus providing no additional contribution to specimen polarisation^29^,^30^. Ca off-centering was experimentally observed by EXAFS in CaTiO_3_^34^ and Ba_1-x_Ca_x_TiO3 (0 < x < 0.5)^30^, by XANES in Ba_1-x_Ca_x_TiO_3_ (x = 0.02, 0.05)^36^ compounds and theoretically predicted in Ba_0.875_Ca_0.125_TiO_3_^24^ and Ba_1-x_Ca_x_TiO_3_^35^. If Ca off-center displacements occur in the same direction as TiO_6_ distortions this additional contribution to polarization results in the increased values of *T*_C_. The direction of Ca off-centering were reported along [111]^30^, [001]^35^,^36^ or [113]^24^ directions. Furthermore Ca displacements were shown to facilitate Ti displacements inside TiO_6_ octahedra further enhancing ferroelectic behaviour^30^. It is possible to speculate that the increase of A-site cation disorder (expressed as σ^2^) could facilitate the shift of smaller A-site cations from the centrosymmetric positions in order to relieve an associated bond strain. A model was proposed that assumed two competitive effects active during Ca doping in Ba_1-x_Ca_x_TiO_3_: the shrinkage of TiO_6_ octahedra resulting in smaller Ti displacements (and possibly away from the c-axis direction) and the increase of the number of off-centered Ca ions. This mode described experimentally observed *T*_C_ dependence reasonably well^35^. Similar A-site driven ferroelectricity was found in perovskites with the t < 1 where the introduction of smaller ions (e.g. Li in K_0.5_Li_0.5_NbO_3_^37^ and Lu in (La,Lu)MnNiO_6_^38^ stabilized off-centering of A-site ion (thus inducing ferroelectric state) over tilting of BO_6_ octahedra^3^.

The observed increase of *T*_*C*_ with σ^2^ and decrease of *T*_*C*_ with <*r*_*A*–*site*_> are in agreement with previous results^16^. A weak increase of γ with 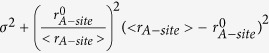
was observed (inset Fig. 3) presumably due to the increased degree of cation disorder. In order to evaluate whether this simple empirical equation (5) consisting of tabulated values of ionic radii, could be used for the selection of compositions with specific values of *T*_*C*_, two compounds (series C) were prepared with an intended *T*_*C*_ of 35 °C. The Ca doping on A site, y, in Ba_1-x-y_Sr_x_Ca_y_TiO_3_ was fixed at y = 0 and 0.1, respectively. A good agreement between the intended and measured values of *T*_*C*_ were observed (31 °C for 0 and 37 °C for 0.1 Ca doped samples).

**Figure 4:**
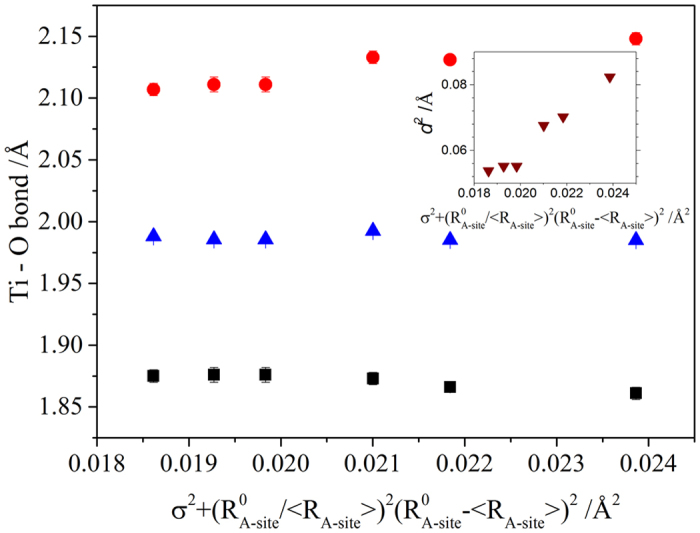
Ti-O bond lengths as a function of 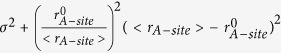. Inset shows d^2^ as function of 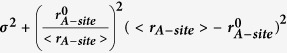.

Neutron diffraction data were collected at room temperature on several samples using the high resolution powder diffractometer, HRPD, at the ISIS neutron facility, Rutherford Appleton Laboratories, UK. Diffraction patterns were recorded over the time-of-flight range 31–125 ms, corresponding to a *d*-spacing range 0.65–2.58 Å, or 0.85–3.89 Å, for patterns collected in the back-scattering and 90 degree detector banks, respectively. The patterns were recorded to a total incident proton beam of about 60 μA h. The neutron patterns from back scattered and 90 degree banks were fitted simultaneously by Rietveld profile refinement method using Gsas II software^39^. The data were refined in P4 mm space group with the following atomic positions: Ti (0, 0, 0), Ba/Sr/Ca (0.5, 0.5, z), O1 (0, 0, z), O2 (0, 0.5, z). Due to strong correlation, atomic positions and isotropic thermal factors for A-site cations were not refined independently. As a result we were unable to model Ca ion off-centering ions as discussed above. The cation occupancies were fixed according to the results of the chemical analysis and the full oxygen occupancy was assumed. The following parameters were refined: background coefficients, scale factors, diffractometer constant, peak shape, anisotropic strain, atomic positions and isotropic displacement parameters. The results of the refinements are given in the Table 3. As a function 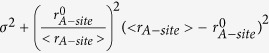 increased, one Ti-O1 bond monotonically increased whereas another Ti-O1 bond decreased leading to the distortion of TiO_6_ octahedra (Fig. 4). As a result a linear increase of the squared atomic displacement of Ti ions from the centrosymemtric position, *d*^2^, is observed (inset Fig. 4).

**Figure 5:**
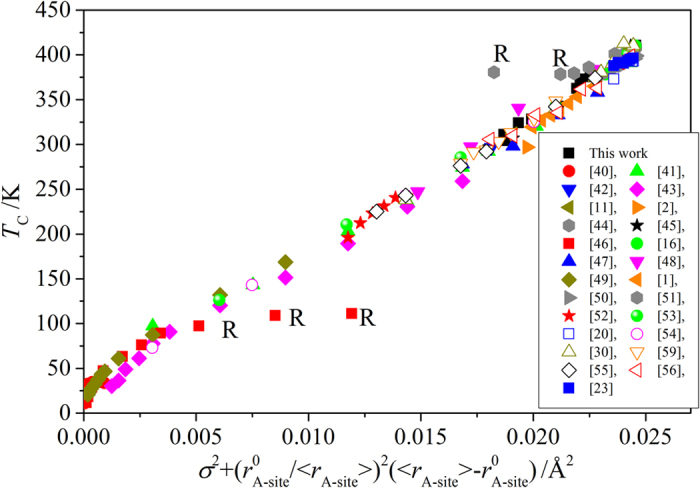
*T*_*C*_ as a function of 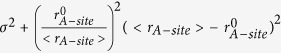 for samples studied in this work and reported in the literature. R denotes samples with relaxor type behavior.

Figure 5 shows the *T*_C_ as a function of 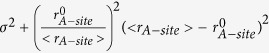 in a large number of Ba_1-x-y_Sr_x_Ca_y_TiO_3_ with the tolerance factor greater than 1 reported in the literature over the last 60 years^12^,^20^,^40^,^41^,^42^,^43^,^44^,^45^,^46^,^47^,^48^,^49^,^50^,^51^,^52^,^53^,^54^,^55^,^56^,^57^,^58^,^59^. A strong linear correlation is observed with some scatter of data presumably due to compositional inhomogeneity (including partial substitution of Ca on B site^52^), annealing conditions^5^, thermal hysteresis during the measurements^43^, etc. In datasets^44^,^46^ that showed a deviation from the observed empirical trend, frequency dependences of ε_r_ was observed in heavily doped Ca samples. As a result we suggest that the observed empirical equation (5) is valid for typical ferroelectrics.

**Figure 6:**
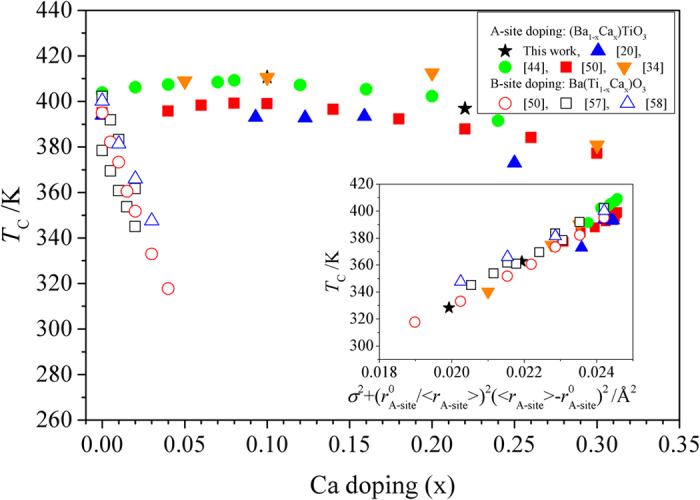
*T*_C_ as a function of Ca doping and 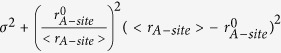 (inset).

It is interesting to consider the effect of Ca doping on *T*_C_ in Ba_1-y_Ca_y_TiO_3_ compounds. It has been shown that Ca doping on A-site results in first slight increase of *T*_C_ with up to 8% Ca doping followed by a decrease^20^,^30^,^44^,^51^, whereas even small extend of Ca doping on B site resulted in a drastic decrease of *T*_C_^51^,^57^,^58^ (Fig. 6). From the proposed model the introduction of smaller Ca ions in BaTiO_3_ resulted in the increase of σ^2^ and the decrease of 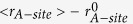 thus exhibiting opposite effects on the values of *T*_C_. As a result *T*c is expected to show the maximum with Ca doping. When the *T*_C_ data was replotted as a function of 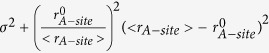 a monotonic increase of *T*_C_ was observed (inset to Fig. 6) regardless of Ca doping on A- or B-sites (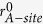 increased with Ca doping on the Ti-site). Furthermore our preliminary results showed that the correlation similar to (5) exists in Zr and Sn doped titanates. As a result it is possible that the observed empirical correlation (5) is valid for the families of titanates with A- and B-sites doped by isoelectronic ions. At the same time the proposed model is expected to break down when the ion shape cannot be considered spherical, for example when the stereochemically active electron lone pairs (Pb^2+^, Bi^3+^) or partially filled orbitals (La^3+^) are present as shown in Supplementary Fig. S7.

**Figure 7:**
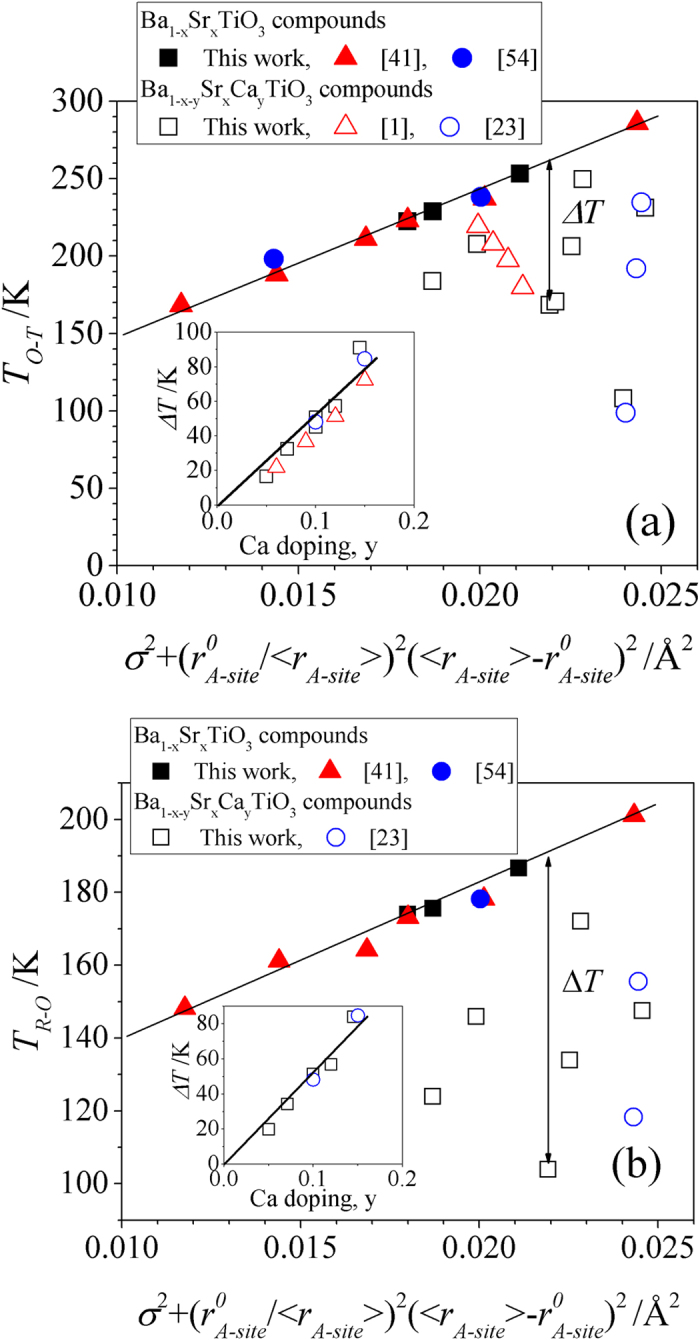
Temperatures of orthorhombic to tetragonal transition, *T*_*O-T*_, (**a**) and rhombohedral to orthorhombic transition, *T*_*R-O*_, (**b**) as function of 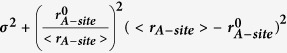. The temperature deviation, Δ*T*, of Ca containing samples (Ba_1-x-y_Sr_x_Ca_y_TiO_3_, open symbols) from line for only Ba, Sr containing compounds (Ba_1-x_Sr_x_TiO_3_, solid symbols). Insets show Δ*T* as a function of Ca doping, y. Solid lines are guides to eye.

The temperatures for rhombohedral to orthorhombic, *T*_*R-O*_, and orthorhombic to tetragonal, *T*_*O-T*_, transitions in BSCT are shown in Fig. 7(a,b), respectively. For compounds containing only Ba and Sr on the A-site, a linear increase of the transition temperatures with 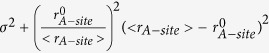 was observed. The Ca containing samples showed negative deviation of *T*_*R-O*_ and *T*_*O-T*_ from the linear trends of Ba_1-x_Sr_x_TiO_3_ compounds (marked as Δ*T* on Fig. 7(a,b)). Δ*T* increased linearly with Ca doping, y, regardless of Sr and Ba content (insets to Fig. 7 (a,b)). The following empirical equations were proposed:

for orthorhombic to tetragonal transition (y < 0.2)


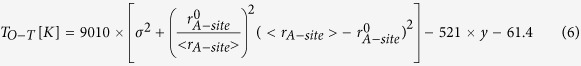


for rhombohedral to orthorhombic transition (y < 0.15)


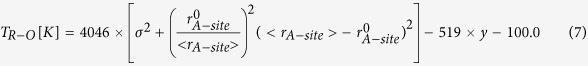


In conclusion we demonstrated a combined effect of average ionic radii and cation variance on *T*_*C*_ a Ba_1-x-y_Sr_x_Ca_y_TiO_3_ ferroelctric perovskites. *T*_*C*_ increased linearly with σ^2^ and <*r*_*A*–*site*_>. A set of empirical equations was proposed which allowed the estimation of temperature of phase transitions in alkaline-earth titanates based on the tabulated values of ionic radii. This provides simple guidelines for a selection of compounds with required values phase transitions temperatures (*T*_*C*_, *T*_*R-O*_ and *T*_*O-T*_) in Ba_1-x-y_Sr_x_Ca_y_TiO_3_ perovskites.

## Additional Information

**How to cite this article**: Berenov, A. *et al*. Effect of ionic radii on the Curie temperature in Ba_1-x-y_Sr_x_Ca_y_TiO_3_ compounds. *Sci. Rep.* **6**, 28055; doi: 10.1038/srep28055 (2016).

## Supplementary Material

Supplementary Information

## Figures and Tables

**Table 1:** Crystallographic parameters of studied samples.

Composition	< *r*_*A*–*site*_> (Å)	*δ*^2^ (Å^2^)	*a* (Å)	*c* (Å)	*c/a*
Series A					
Ba_0.65_Sr_0.35_TiO_3_	1.5505	0.0066	3.9657(1)	3.9739(1)	1.0021
Ba_0.69_Sr_0.24_Ca_0.07_TiO_3_	1.5505	0.0085	3.9655(4)	3.9831(8)	1.0044
Ba_0.74_Sr_0.12_Ca_0.15_TiO_3_	1.5506	0.0105	3.9633(6)	3.9938(8)	1.0077
Ba_0.78_Ca_0.22_TiO_3_	1.5506	0.0125	3.9631(7)	4.0066(9)	1.0110
Series B					
Ba_0.8_Sr_0.2_TiO_3_	1.5760	0.0046	3.9804(1)	4.0045(4)	1.0061
Ba_0.6_Sr_0.2_Ca_0.2_TiO_3_	1.5220	0.0126	3.9500(1)	3.9640(5)	1.0036
Ba_0.9_Ca_0.1_TiO_3_	1.5830	0.0066	3.9784(1)	4.0236(1)	1.0114
Ba_0.85_Sr_0.1_Ca_0.05_TiO_3_	1.5795	0.0056	3.9793(2)	4.0149(2)	1.0089
Ba_0.78_Sr_0.1_Ca_0.12_TiO_3_	1.5606	0.0092	3.9696(2)	4.0043(2)	1.0088
Ba_0.75_Sr_0.1_Ca_0.15_TiO_3_	1.5525	0.0105	3.9609(2)	3.9956(3)	1.0088
Ba_0.7_Sr_0.1_Ca_0.2_TiO_3_	1.5390	0.0124	3.9576(2)	3.9884(2)	1.0078
Series C					
Ba_0.68_Sr_0.32_TiO_3_	1.5559	0.0063	3.9688(1)	3.9795(3)	1.0027
Ba_0.62_Sr_0.28_Ca_0.1_TiO_3_	1.5359	0.0098	3.9580(1)	3.9683(4)	1.0026

**Table 2:** The results of dielectric measurements of studied compounds.

Composition	*T*_*R-O*_ (K)	*T*_*O-T*_ (K)	*T*_*C*_ (K)	*ε*_*rm*_	*T*_*0*_ (K)	*γ*
Series A						
Ba_0.65_Sr_0.35_TiO_3_	174	222	294	2566	271	1.09
Ba_0.69_Sr_0.24_Ca_0.07_TiO_3_	146	208	328	2635	289	1.03
Ba_0.74_Sr_0.12_Ca_0.15_TiO_3_	104	167	363	6132	351	1.12
Ba_0.78_Ca_0.22_TiO_3_	NA	NA	397	4049	373	1.19
Series B						
Ba_0.8_Sr_0.2_TiO_3_	187	253	343	4483	322	1.11
Ba_0.6_Sr_0.2_Ca_0.2_TiO_3_	NA	NA	324	2692	301	1.18
Ba_0.9_Ca_0.1_TiO_3_	148	231	411	2994	385	1.27
Ba_0.85_Sr_0.1_Ca_0.05_TiO_3_	172	250	378	4251	365	1.15
Ba_0.78_Sr_0.1_Ca_0.12_TiO_3_	134	206	375	3697	360	1.21
Ba_0.75_Sr_0.1_Ca_0.15_TiO_3_	NA	170	373	3161	357	1.25
Ba_0.7_Sr_0.1_Ca_0.2_TiO_3_	NA	94	363	3826	353	1.24
Series C						
Ba_0.68_Sr_0.32_TiO_3_	176	229	304	3737	269	1.08
Ba_0.62_Sr_0.28_Ca_0.1_TiO_3_	124	184	311	9564	309	1.18

**Table 3:** Refined parameters obtained from Rietveld refinements on neutron data.

Parameter	Series A			Series B		Series C
	Ba_0.69_Sr_0.24_Ca_0.07_TiO_3_	Ba_0.74_Sr_0.12_Ca_0.15_TiO_3_	Ba_0.78_Ca_0.22_TiO_3_	Ba_0.8_Sr_0.2_TiO_3_	Ba_0.6_Sr_0.2_Ca_0.2_TiO_3_	Ba_0.68_Sr_0.32_TiO_3_
a (Å)	3.96435(3)	3.96279(3)	3.96231(7)	3.97818(4)	3.94981(4)	3.96849(3)
c (Å)	3.98716(5)	3.99661(4)	4.00921(9)	4.00558(6)	3.97230(7)	3.98159(4)
z (A-site)	0.514(2)	0.512(14)	0.509(2)	0.512(2)	0.511(3)	0.519(2)
Uiso(A-site) (Å^2^)	0.0056(2)	0.0097(2)	0.0073(3)	0.0079(2)	0.0106(2)	0.0070(2)
Uiso (Ti) (Å^2^)	0.0033(4)	0.0071(3)	0.0050(4)	0.0071(4)	0.0064(5)	0.0042(4)
z (O1)	0.530(2)	0.5332(1)	0.536(1)	0.527(1)	0.529(2)	0.529(1)
Uiso (O1) (Å^2^)	0.0079(6)	0.0115(4)	0.0100(5)	0.0098(5)	0.0140(7)	0.0093(5)
z (O2)	0.029(1)	0.030(1)	0.031(1)	0.029(1)	0.029(2)	0.031(1)
Uiso (O2) (Å^2^)	0.0058(3)	0.0101(2)	0.0080(3)	0.0087(3)	0.0095(3)	0.0071(3)
wRp	4.11	4.34	5.90	4.17	4.11	4.15
Ti-O1 (Å)	1.876(6)	1.866(4)	1.861(5)	1.873(5)	1.870(7)	1.875(5)
Ti-O1 (Å)	2.111(6)	2.131(4)	2.148(5)	2.133(5)	2.102(7)	2.107(5)
Ti-O2 (Å)	1.9856(3)	1.9851(2)	1.9850(3)	1.9924(3)	1.9782(4)	1.9881(3)
Ti-O-Ti (°)	173.2(3)	173.0(2)	172.9(3)	173.4(3)	173.4(4)	172.9(2)
A-O1 (Å)	2.8039(1)	2.8034(1)	2.8039(2)	2.8143(1)	2.7938(1)	2.8604(7)
A-O2 (Å)	2.767(3)	2.762(3)	2.756(3)	2.774(3)	2.753(4)	2.778(3)
A-O2 (Å)	2.857(4)	2.862(3)	2.882(4)	2.872(3)	2.850(4)	2.844(3)
