# Supplementary material for: Effect of ionic radii on the Curie temperature in Ba1-x-ySrxCayTiO3 compounds
Source: Sci Rep. 2016 Jun 21;6:28055. doi: 10.1038/srep28055 (PMC4914972; doi:10.1038/srep28055)
Supplement: Supplementary Information [file srep28055-s1.pdf]

Effect of ionic radii on the Curie temperature in $Ba_{1-x-y}Sr_xCa_yTiO_3$ compounds

A. Berenov,[a] F. Le Goupil[a] and N. Alford[a]

[a] Department of Materials, Imperial College London, London, SW7 2AZ,UK.

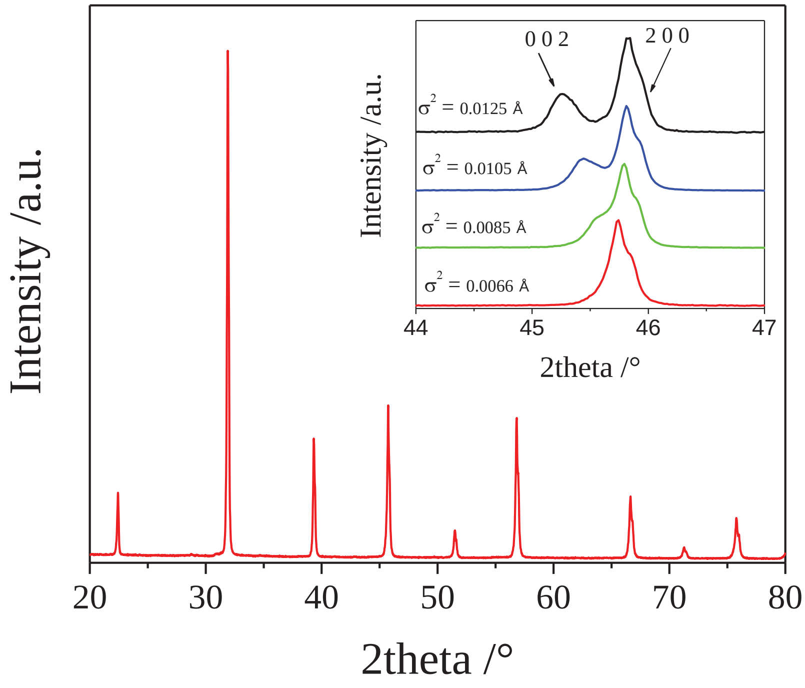

Supplementary Figure S1. XRD pattern of $Ba_{0.65}Sr_{0.35}TiO_3$. The inset shows tetragonal splitting of (200) peak in series A compounds.

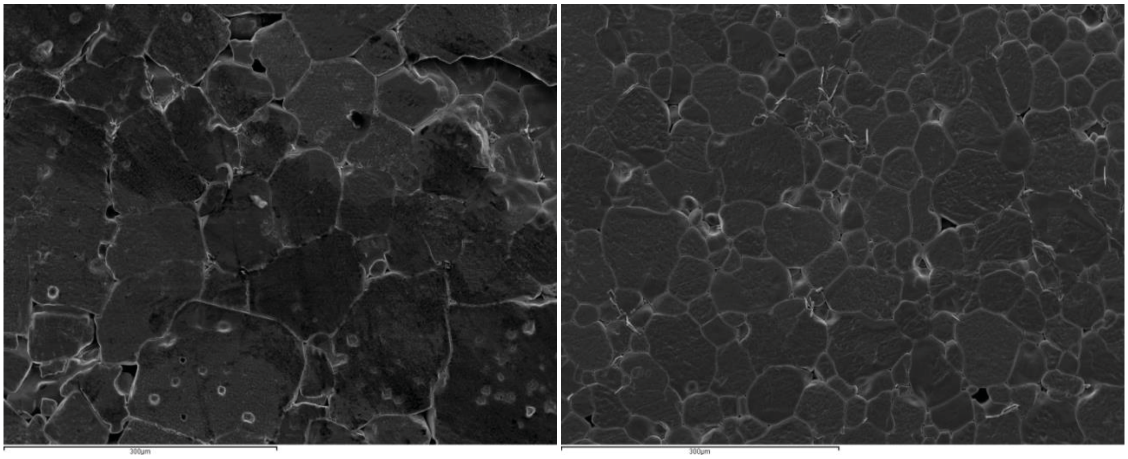

(a) (b)

Supplementary Figure S2. SEM images of $Ba_{0.65}Sr_{0.35}TiO_3$ (a) and $Ba_{0.78}Ca_{0.22}TiO_3$ (b) pellets thermally etched at 1400 °C for 0.5 hrs

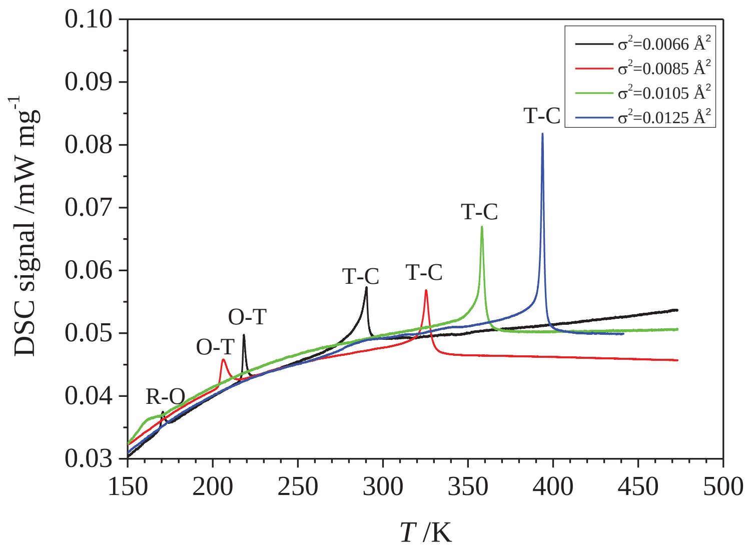

Supplementary Figure S3. Temperature dependences of DSC signal for compounds from series A.

Supplementary Table S4 ICP and DSC results of studied BSCT samples.

Intended composition	Analysed composition	$T_{R\text{-}O}$ /K	$T_{O\text{-}T}$ /K	T_C /K
Series A				
$Ba_{0.65}Sr_{0.35}TiO_3$	$Ba_{0.655(2)}Sr_{0.349(1)}TiO_3$	171	218	291
$Ba_{0.69}Sr_{0.24}Ca_{0.07}TiO_3$	$Ba_{0.704(2)}Sr_{0.237(1)}Ca_{0.076(1)}TiO_3$	145	206	325
$Ba_{0.74}Sr_{0.12}Ca_{0.15}TiO_3$	$Ba_{0.742(14)}Sr_{0.120(2)}Ca_{0.144(1)}TiO_3$	NA	163	358
$Ba_{0.78}Ca_{0.22}TiO_3$	$Ba_{0.798(9)}Ca_{0.206(1)}TiO_3$	NA	NA	394
Series B				
$Ba_{0.8}Sr_{0.2}TiO_3$	$Ba_{0.796(3)}Sr_{0.197(1)}TiO_3$	187	251	341
$Ba_{0.6}Sr_{0.2}Ca_{0.2}TiO_3$	$Ba_{0.607(6)}Sr_{0.202(3)}Ca_{0.198(1)}TiO_3$	NA	NA	320
$Ba_{0.9}Ca_{0.1}TiO_3$	$Ba_{0.887(1)}Ca_{0.113(2)}TiO_3$	139	224	402
$Ba_{0.85}Sr_{0.1}Ca_{0.05}TiO_3$	$Ba_{0.854(2)}Sr_{0.100(1)}Ca_{0.046(1)}TiO_3$	173	248	376
$Ba_{0.78}Sr_{0.1}Ca_{0.12}TiO_3$	$Ba_{0.794(2)}Sr_{0.103(1)}Ca_{0.103(1)}TiO_3$	NA	NA	369
$Ba_{0.75}Sr_{0.1}Ca_{0.15}TiO_3$	$Ba_{0.754(2)}Sr_{0.101(1)}Ca_{0.145(1)}TiO_3$	NA	NA	363
$Ba_{0.7}Sr_{0.1}Ca_{0.2}TiO_3$	$Ba_{0.704(1)}Sr_{0.102(1)}Ca_{0.195(1)}TiO_3$	NA	NA	357
Series C				
$Ba_{0.68}Sr_{0.32}TiO_3$	$Ba_{0.692(3)}Sr_{0.321(6)}TiO_3$	176	227	302
$Ba_{0.62}Sr_{0.28}Ca_{0.1}TiO_3$	$Ba_{0.625(3)}Sr_{0.283(4)}Ca_{0.098(1)}TiO_3$	NA	180	307

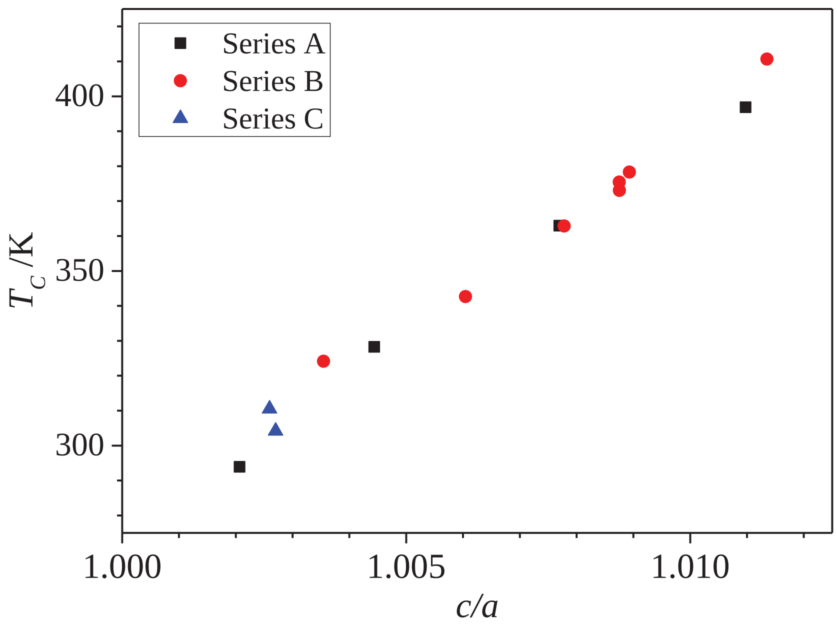

Supplementary Figure S5. T_C as a function of *c/a* for the studied compounds.

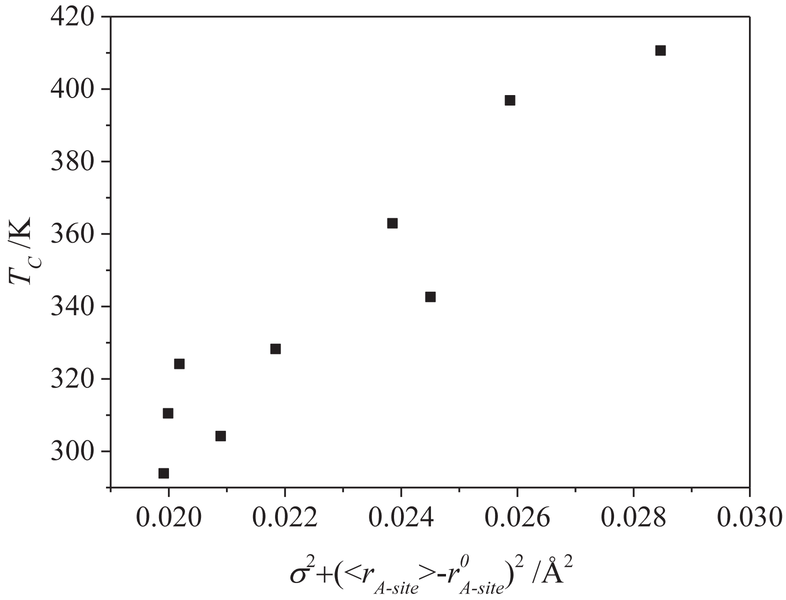

Supplementary Figure S6. T_C as a function of $\sigma^2+\left(<r_{A-site}>-r^0_{A-site}\right)^2$ for samples studied in this work.

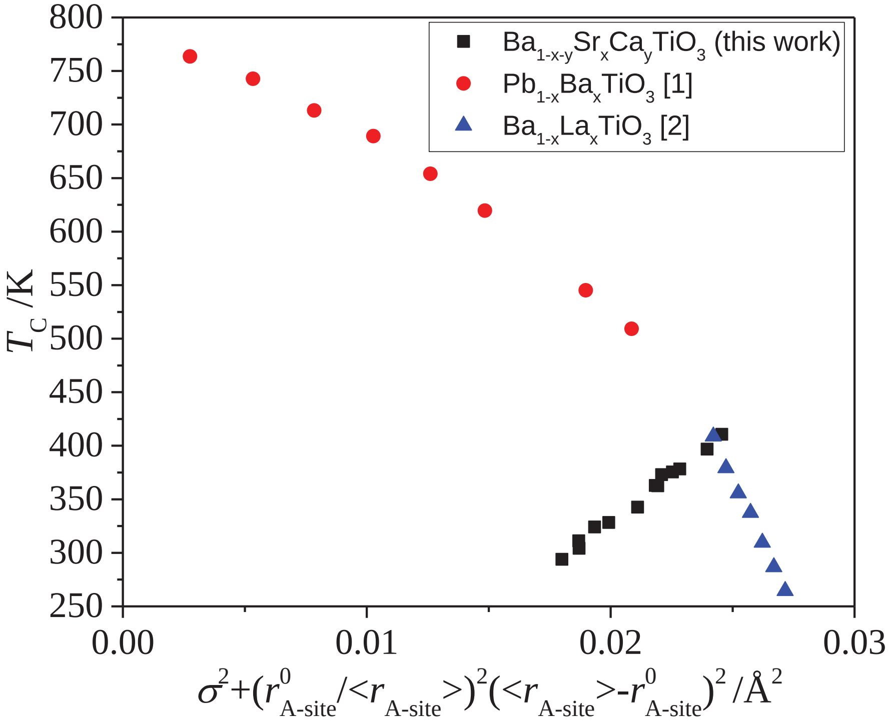

Supplementary Figure S7. T_C as a function of $\sigma^2 + \left(\frac{r^0_{A-site}}{<r_{A-site}>}\right)^2 (< r_{A-site} > -r^0_{A-site})^2$ for titanates with isoelectronic ($Ba_{1-x-y}S_xCa_yTiO_3$ this work) and non-isoelectronic ($Pb_{1-x}Ba_xTiO_3$ [1], $Ba_{1-x}La_xTiO_3$[2]) doping on the A site.

---

[1] T. Ikeda, J. Phys. Soc. Jap. **14** 1286 (1959)

[2] L. Ben and D. C. Sinclair, Appl. Phys. Lett., **98** 092907 (2011)
